# Integrity of the Antiviral STING-mediated DNA Sensing in Tumor Cells Is Required to Sustain the Immunotherapeutic Efficacy of *Herpes Simplex* Oncolytic Virus

**DOI:** 10.3390/cancers12113407

**Published:** 2020-11-17

**Authors:** Guendalina Froechlich, Carmen Caiazza, Chiara Gentile, Anna Morena D’Alise, Maria De Lucia, Francesca Langone, Guido Leoni, Gabriella Cotugno, Vittorio Scisciola, Alfredo Nicosia, Elisa Scarselli, Massimo Mallardo, Emanuele Sasso, Nicola Zambrano

**Affiliations:** 1 CEINGE Biotecnologie Avanzate S.C.aR.L., Via G. Salvatore 486, 80145 Naples, Italy; guendalina.froechlich@unimi.it (G.F.); chiara.gentile@unina.it (C.G.); v.scisciola@studenti.unina.it (V.S.); alfredo.nicosia@unina.it (A.N.); zambrano@unina.it (N.Z.); 2 Department of Molecular Medicine and Medical Biotechnology (DMMBM), University of Naples Federico II, Via Pansini 5, 80131 Naples, Italy; carmen.caiazza@unina.it (C.C.); massimo.mallardo@unina.it (M.M.); 3 Nouscom S.R.L., Via di Castel Romano 100, 00128 Rome, Italy; m.dalise@nouscom.com (A.M.D.); m.delucia@nouscom.com (M.D.L.); f.langone@nouscom.com (F.L.); G.Leoni@nouscom.com (G.L.); g.cotugno@nouscom.com (G.C.); E.Scarselli@nouscom.com (E.S.)

**Keywords:** *Herpes simplex*, HSV-1, oncolytic virus, immunogenic cell death, STING knockout, RNA profiling, MB21D, TMEM173

## Abstract

**Simple Summary:**

Oncolytic viruses are emerging immunotherapeutics in cancer treatments. The conflicting role of innate immunity in the antitumor activity of oncolytic viruses is still a matter of debate. The STING-dependent DNA sensing axis is considered detrimental for viral replication and cancer cell clearance. Accordingly, we observed that STING loss in tumor cells was associated with improved lytic potential by a herpes-based oncolytic virus. However, STING-knockout cancer cells infected with the oncolytic virus showed impaired immunogenicity, as immunogenic cell death was improperly triggered. In agreement with these observations, STING-knockout tumors raised in a murine syngeneic model were more resistant to a combined treatment of the oncolytic virus with PD-1 blockade. The present study demonstrates the antitumor benefit of antiviral immunity and sheds lights on the mechanisms of immune resistance to oncovirotherapy exerted by STING-loss in tumor cells.

**Abstract:**

The dichotomic contribution of cancer cell lysis and tumor immunogenicity is considered essential for effective oncovirotherapy, suggesting that the innate antiviral immune response is a hurdle for efficacy of oncolytic viruses. However, emerging evidence is resizing this view. By sensing cytosolic DNA, the cyclic GMP-AMP synthase (*cGAS*) and stimulator of interferon genes (*STING*) axis can both counteract viral spread and contribute to the elicitation of adaptive immunity via type I interferon responses. In this paper, we analyzed the tumor-resident function of *Sting*-mediated DNA sensing in a combined approach of oncovirotherapy and PD-1 immune checkpoint blockade, in an immunocompetent murine model. While supporting increased lytic potential by oncolytic HER2-retargeted HSV-1 in vitro and in vivo, *Sting*-knockout tumors showed molecular signatures of an immunosuppressive tumor microenvironment. These signatures were correspondingly associated with ineffectiveness of the combination therapy in a model of established tumors. Results suggest that the impairment in antiviral response of *Sting*-knockout tumors, while favoring viral replication, is not able to elicit an adequate immunotherapeutic effect, due to lack of immunogenic cell death and the inability of *Sting*-knockout cancer cells to promote anti-tumor adaptive immune responses. Accordingly, we propose that antiviral, tumor-resident *Sting* provides fundamental contributions to immunotherapeutic efficacy of oncolytic viruses.

## 1. Introduction

Oncolytic viruses (OV) are modified or naturally occurring viral particles able to selectively kill cancer cells [1], inducing tumor immunogenicity [2]. Combination therapies of OVs with immune checkpoint inhibitors (ICI) show additive or even synergistic effects overcoming the resistance to PD-1 and CTLA-4 blockade [2,3,4,5]. The immunogenic way in which cancer cells succumb to oncolytic viruses is actually the essence of their immunotherapeutic behavior. Immunogenic cell death (ICD) consists of the release of damage-associated molecular patterns (DAMPs e.g., HMGB1, ATP, Type I interferons) accompanied by tumor-associated antigens (TAA) and tumor-specific antigens (TSA) that are captured and presented by antigen-presenting cells (APCs) [6,7].

The identification of the best balance between oncolytic and immunotherapeutic activity, that could allow us to take full advantage of oncovirotherapy, is far to be fully elucidated and is still a matter of debate. In this regard, the innate antiviral response to OV is the key point, as on one side it may result in being detrimental for intratumor viral replication while, on the other side, it should trigger the desired inflammation within the tumor microenvironment (TME) [8]. Within this scenario, the pattern-recognition receptors (PRRs) are the key molecular determinants that recognize pathogen-associated molecular patterns (PAMPs) and trigger type I interferons (IFNs) cascade [8]. Recently, the cyclic GMP-AMP synthase (cGAS) has been identified as a cytosolic DNA sensor able to activate stimulator of interferon genes (STING) by 2′,3′-cGAMP, triggering TANK binding kinase 1 (TBK1)-IFN regulatory factor 3 (IRF3) axis and type I IFN response [9].

The *STING* pathway efficiently counteracts viral infection by both inhibiting in-cell viral replication and protecting surrounding non-infected cells from viral spread through: (i) paracrine type I IFNs secretion; (ii) cGAMP transfer via gap junctions; (iii) release of extracellular vesicles and cell debris containing activated STING and cGAMP [9,10,11,12]. This is true not only for DNA viruses including adenoviruses, poxviruses and *Herpes* viruses, but also for RNA viruses (e.g., VSV) [13]. Simultaneously, under co-evolutionary selective pressure, many viruses, including HSV-1, have developed the ability to escape the *STING* pathway at each signaling cascade level [14,15,16].

STING agonists are currently being evaluated in clinical trials (NCT02675439, NCT03937141) for cancer treatment, since the *cGAS–STING* axis emerged as essential to activate antitumor effector T cells in response to genotoxic stresses and immune-based therapies [17,18,19,20,21,22].

Even though the essential role of *STING* in immune cells has been largely clarified, its tumor-resident function is still largely unexplored. Analysis of TCGA database shed light on this tumor-intrinsic role, since loss-of-function mutations and epigenetic silencing occur in carcinomas across the *cGAS-STING-TBK1-IRF3* axis, with a higher inactivation rate, the more advanced the tumor is [23,24,25,26]. The loss-of-function in the antiviral *STING* pathway is thus considered a potential Achilles’ heel of cancer cells that makes them more susceptible to oncolytic viruses, including HSV-1. This enhanced susceptibility has been demonstrated both in vitro, and in immunodeficient mice bearing human tumor xenografts, where OVs induced considerable shrinkage of *STING*-deficient tumors [25,26].

Despite this evidence supporting the application of oncovirotherapy to a wider spectrum of cancer indications, the role of innate antiviral response in establishing adaptive antitumor immunity needs to be evaluated in immunocompetent models. In this field, recent evidence demonstrates that STING^low^ (knock down) compared to STING^high^ cancer cells are slightly more susceptible to lysis and still succumb in an immunogenic way following treatment with T-VEC [27,28], but the complete loss of *STING* that often occurs in cancer cells needs to be assessed.

Based on the aforementioned evidence, in the context of a combination therapy based on oHSV-1 and PD-1 blockade, we aimed to dissect the actual contribution of cancer cell lysis vs. induction of antiviral immune response. Impairment in the antiviral *STING*-mediated DNA sensing was exploited to solve the paradox of improved cancer cell lysis at the expense of immunogenicity in response to oHSV-1. With the aim to dissect the influence of the tumor-resident STING without the interference of tumor-extrinsic contribution of viral DNA sensing, we took advantage from a retargeted *Herpes* virus that is able to selectively infect tumor cells [29]. Thus, we engineered and characterized murine *Sting*-knockout cancer cells, which were also rendered suitable to selective infection by a human HER2-retargeted oHSV-1. Regression of established *Sting*-knockout tumors was evaluated in syngeneic, hHER2-tolerant, immunocompetent mice in the response to combined virotherapy and PD-1 blockade. Molecular analysis of the resected tumors was compatible with the decreased susceptibility of *Sting* knockout tumors to the therapeutic combination. These data support a reappraisal for the use of oncolytic viruses in *STING*-null tumors and underlines the relevance of innate antiviral response to OVs.

## 2. Results

### 2.1. Setup of a Cellular System to Dissect Cancer Cell-Resident STING Pathway In Vitro and In Vivo

With the aim to dissect in vivo the role of the cancer cell-resident *STING* pathway within the tumor microenvironment, we took advantage from the retargeted HSV-1 based oncolytic virus R-LM113, which is able to selectively infect cells expressing the human HER2 receptor and, at the same time, is de-targeted from the natural cellular ligands [29]. Thus, R-LM113 may infect engineered murine tumor cells bearing human HER2, while sparing the cellular components within the tumor microenvironment. This is due to the replacement of the viral glycoprotein D moiety, normally involved in the interaction with host cells via endogenous ligands (HVEM, Nectin-1), with an antibody fragment targeting human HER2. CT26 and LLC1 cell lines derived, respectively, from BALB/c and C57BL/6 murine backgrounds, were selected as tumor models for initial characterizations [30].

**Figure 1:**
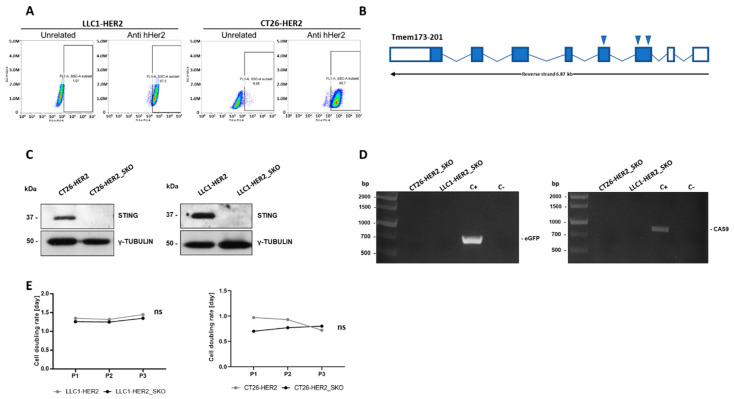
Molecular characterization of *Sting* knockout cancer cell lines. (**A**) Analysis of human HER2 display on cell surface of LLC1-HER2 (left) and CT26_HER2 (right) by FACS analysis; an unrelated antibody was used as negative control. (**B**) The graphic shows *Tmem173* (transcript ID ENSMUST00000115728.4) gene organization. Full and empty boxes represent, respectively, coding and untranslated exons. The positions of guide RNAs used for CRISPR/Cas9 genome editing to generate *Sting* knockout cancer cell lines are indicated by arrows. (**C**) Western blot analysis of Sting protein in CT26-HER2, LLC1_HER2 and their *Sting* knockout cell lines counterparts. Gamma tubulin was used as standard. (**D**) PCR screening of CT26-HER2_SKO and LLC1_HER2_SKO cell lines to assess the absence of eGFP and Cas9 residues in genomic DNA. Cas9/eGFP-encoding vector was used as positive control (C+). Genomic DNA from parental CT26-HER2 and LLC-HER2 cell lines was used as negative control (C−). (**E**) Cell doubling per day were assessed for *Sting* wild-type (grey lines) and *Sting* knockout (black lines) LLC1 (left) and CT26 (right) cell lines. The differences in cell doubling were calculated by Student’s *t*-test and were not statistically significant (Ns) to each passage.

First, we verified that key genes mediating DNA sensing were non-mutated in both LLC1 and CT26 cell lines. In addition, RNA sequencing analysis confirmed an abundant expression of genes involved in cytosolic DNA sensing in both CT26 and LLC1 cell lines, as calculated by Transcripts Per Kilobase Million (Figures S1 and S2). As expected, in the absence of cytoplasmic DNA stimuli, the expression of type I IFNs resulted in the off-state in both cell lines. To allow targeted R-LM113 infection, CT26 cells were stably transduced with the human *HER2* cDNA to generate CT26-HER2 cells, similarly to a LLC1-HER2 cell line, which was already available [31]. The correct display of human HER2 on the cell surfaces of both cell lines was confirmed by FACS analysis (Figure 1A). Thus, *Sting* KO clones were generated by CRISPR/Cas9 genome editing, for both LLC1-HER2 and CT26-HER2 cell lines, hereinafter referred to as CT26-HER2_SKO and LLC1-HER2_SKO. Bona fide targeting was confirmed by Sanger sequencing of the *Sting locus*, which revealed the presence of a deletion and a premature termination codon as result of non-homologous end joining DNA repair (Figure 1B). Accordingly, the Western blot analysis shown in Figure 1C confirmed that all the *Sting* alleles were effectively targeted by Cas9, in the absence of any residual protein expression. The selected knockout clones were also screened by PCR for the absence of potentially immunogenic cloning residues (Cas9, eGFP) hypothetically integrated into the host genome (Figure 1D). *Sting* ablation did not alter the proliferation rate of the parental lines, as assessed by comparison of duplication rates (Figure 1E).

### 2.2. STING Restricts the Replicative Potential of HSV-1 in Cancer Cell Lines

**Figure 2:**
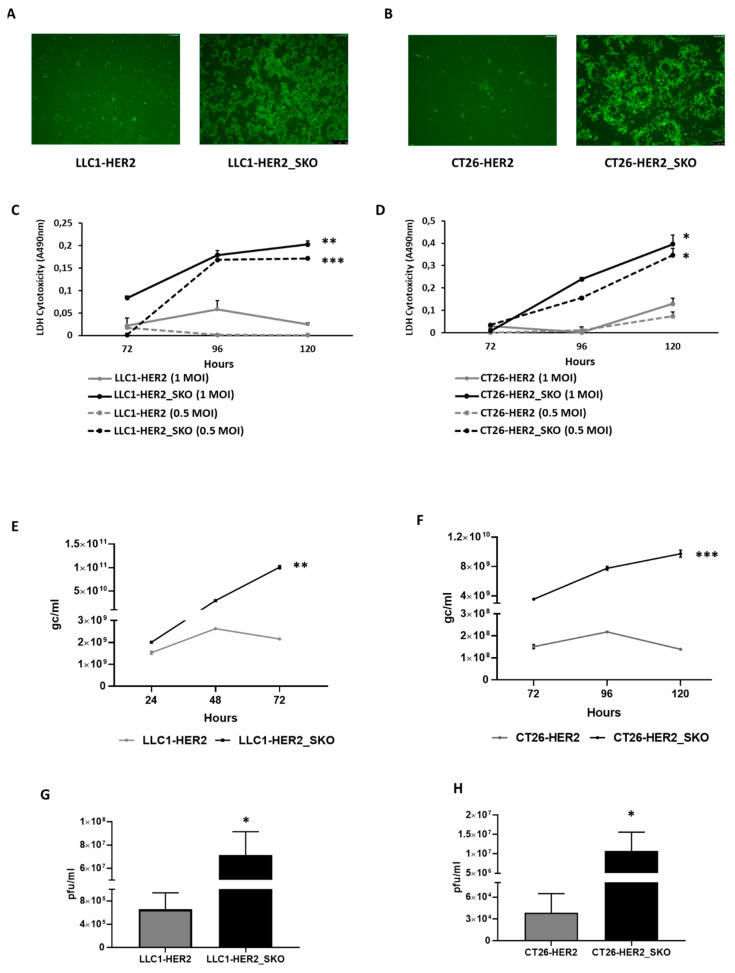
Comparison of viral effectiveness in *Sting* knockout vs. parental wild-type cancer cell lines. (**A**,**B**) Spread of eGFP-encoding R-LM113 was evaluated by fluorescence microscopy in STING wild-type and knockout LLC1 (5×) (**A**) and CT26 (10×) (**B**) cell lines. (**C**) The lytic activity of R-LM113 was evaluated by extracellular LDH (lactate dehydrogenase) release in cell supernatants over the time course of infection (72, 96 and 120 h) in LLC1-HER2 (grey lines) and LLC1-HER2_SKO (black lines) at two different concentrations of viral particles (1 multiplicity of infection (MOI) continuous lines and 0.5 MOI dashed lines). (**D**) The same experiments performed in panel C were recapitulated in CT26-HER2 and CT26-HER2_SKO. All the infections were performed as biological replicates. The statistical significances for experiments described in panel c and d were calculated by Student’s *t*-test comparing MOI-matched *Sting* wild-type vs. knockout cell lines. The *p*-values were 0.00115 and 0.000219, respectively, for 1 and 0.5 MOI in panel C; 0.01583, 0.008543, respectively, for 1 and 0.5 MOI in panel D. (**E**,**F**) Evaluation of viral replication of R-LM113 in *Sting* wild-type and knockout LLC1 (**E**) and CT26 (**F**) infected with 0.3 PFU/cell. The qPCR-TaqMan analysis revealed the genome copies per mL (gc/mL) produced by the virus over time (24, 48, 72 h for LLC1 and 72, 96, 20 h for CT26). The statistical significances for experiments described in panel e and f were calculated by Student’s *t*-test comparing *Sting* wild-type vs. knockout cell lines. The *p*-values calculated on biological replicates were 0.0013 for LLC1 cell line and 0.0005 for CT26 cell line. (**G**,**H**) Analysis of the R-LM113 viral titers obtained in *Sting* wild-type and knockout LLC1 (**G**) and CT26 (**H**) cells infected with 0.3 PFU/cell. Plaque assay was performed as biological replicate. The statistical significance for experiments described in panel g and h was calculated by Student’s *t*-test comparing *Sting* wild-type vs. knockout cell lines. The *p*-values were 0.038 for LLC1 cell line and 0.02 for CT26 cell line. *p* < 0.05 *; *p* < 0.005 **; *p* < 0.0005 ***.

The impact of *Sting* loss-of-function on the impairment of the antiviral response in cancer cells was initially evaluated in vitro by infection of *Sting* KO and wild-type cells with the oncolytic R-LM113 virus. Both CT26-HER2_SKO and LLC1-HER2_SKO cell lines were more susceptible to oncolytic R-LM113, compared to their *Sting* wild-type counterparts. On the contrary, the entry ability of R-LM113 remained unaffected by *Sting* KO (Figure S3). Oncolytic virus spread, assessed by viral-encoded eGFP expression, evidenced the formation of large lysis plaques in *Sting* knockout cells, compared to the viral restraint observed in the *Sting* wild-type statuses of both parental cell lines (Figure 2A,B). Interestingly, as reported in the independent scientific literature [24,25,26,27], we confirmed that the efficiency of viral replication is actually related to Sting expression, since *Sting* knock down cells show an intermediate level of viral replication, compared to *Sting* wild-type and knockout cells (Figure S4).

The lytic activity of oncolytic R-LM113 was also evaluated through extracellular LDH (lactate dehydrogenase) release at different time points after infection; R-LM113 revealed a dose-dependent escalation in cytotoxicity in both CT26-HER2_SKO and LLC1-HER2_SKO cell lines compared to wild-type counterparts (Figure 2, Panels C and D). We thus extended characterizations to viral genome replication and actual production of infective viral particles. Figure 2E,F show the results of viral replication in CT26-HER2_SKO and LLC1-HER2_SKO cell lines, respectively; the functional inactivation of *Sting* exerted a disruptive gain in viral replication in both cell lines. *Sting* loss also overcame the drop in DNA replication observed during the time-course analysis of both parental cells between 96 and 120 h post infection, presumably due to triggering of antiviral responses by *Sting* in wild-type cells. The dramatic increases in viral DNA replication observed after *Sting* loss were accordingly accompanied by correspondingly high increases in viral maturation and production, as shown in the panels g and h of Figure 2, respectively, for CT26-HER2_SKO and LLC1-HER2_SKO cells. These data support the central role of *Sting* in mediating cellular antiviral responses, independently from the genetic background-dependent differential basal susceptibilities of the two cellular systems under analysis. The results obtained with the human HER2-retargeted R-LM113 virus were properly replicated with the R-LM55 virus derived from wild-type strain F HSV-1 (Figure S5), revealing that an entry-independent mechanism is involved in the increased susceptibility of *Sting*-knockout cells to viral activities. 

Thus, the functional inactivation of *Sting* exerted dramatic gains in both viral replication and production, that increased, respectively, by 75- and 250-fold for CT26-HER2_SKO, and by 50- and 100- fold for LLC1-HER2_SKO cells, compared to the matching *Sting* wild-type cell lines. Accordingly, maturation of viral particles was particularly favored by *Sting* inactivation. To further confirm that the gain in viral replication was actually *Sting*-dependent, a functional rescue of Sting activity was carried out in the knockout cells. CT26-HER2_SKO cells were transiently transfected with a *Sting*-encoding vector. The day after transfection, cells were infected with R-LM113 virus, and viral functions were monitored for up to 72 h post infection. The functional rescue of *Sting* completely restored the trend of resistance of CT26-HER2_SKO to viral infection according to both viral spread and viral replication (Figure S6).

These data demonstrate that *Sting* pathway efficiently counteracts the infection of HSV-1, even in the presence of functional viral anti-*Sting* genes (e.g., γ34.5) held by the non-attenuated R-LM113 and R-LM55 viruses [16,27]. Altogether, these data suggest that tumor cells with impaired DNA-sensing *Sting* pathway could potentially represent an improved target for oncolytic virotherapy.

### 2.3. Sting_KO-Dependent Improvements in Oncolytic Viral Replication and Cytotoxicity Do Not Correlate with Tumor Clearance Efficacy In Vivo

**Figure 3:**
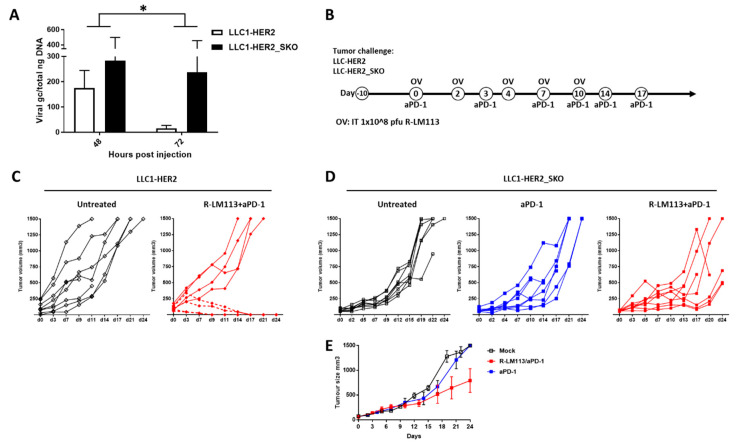
Tumor-resident STING influences oncolytic R-LM113 activity in vivo. (**A**) Evaluation of in vivo intratumoral viral replication in *Sting* wild-type and knockout LLC1 cell lines at 48 and 72 h after administration of R-LM113 (1E+08 viral PFU). Viral genome copies were quantified by TaqMan PCR and were normalized to total ng of extracted DNA. The statistical significance was calculated by two-way ANOVA (0.0148). (**B**) Schematic representation of the in vivo experimental setting. LLC1-HER2 wild-type and knockout cells were implanted subcutaneously into hHER2-transgenic/tolerant mice. When tumors became established (mean 110 mm^3^), mice were randomized according to tumor size. Mice received 5 intratumoral injections of R-LM113 (1E+08 PFU/inj) at 0, 2, 4, 7, 10 days and six systemic administrations of PD-1 blocking antibody at days 0, 3, 7,10, 14, 17. (**C**) LLC-HER2 tumor growth in corresponding untreated (empty rhombuses) and combination treatment (red rhombuses). Dashed lines indicate complete responder mice. R-LM113 and PD-1 blockade monotherapy does not have in vivo efficacy [32,33,34] (**D**) LLC-HER2_SKO tumor growth for the three experimental groups: untreated (empty square), α-mPD-1 (blue) and combination (red square). For c and d, each line represents the tumor growth for individual mouse. The statistical significance for experiments described in panel c was calculated by Fisher’s and was 0.03. (**E**) Median tumor volume with SEM for mice presented in panel d.

After having established that tumor cells with unfunctional *Sting* are more susceptible to oncolytic virus propagation in vitro, we investigated the contribution of cancer-cell-intrinsic, *Sting*-dependent antiviral response in an immunocompetent tumor mouse model. To investigate in which way tumor-intrinsic *Sting* may affect oncolytic efficacy in vivo, we implemented the LLC1 syngeneic, human HER2-tolerant mouse model [31]. First, C57-HER2 tolerant mice were injected subcutaneously with LLC1-HER2 or LLC1-HER2_SKO cells. When tumors became established (approximately 100 mm^3^), mice were treated with a single intra-tumoral injection of R-LM113 virus. The in vivo viral replication was evaluated into the tumors confirming that in vivo, as occurring in vitro, *Sting* knockout tumors sustained a more active viral replication, compared to *Sting* wild-type tumors (Figure 3A).

Since the R-LM113 oncolytic virus has been largely reported to be poorly effective as a single agent in this established tumor setting [32], we evaluated its therapeutic efficacy in combination with PD-1 immune checkpoint inhibition according to the treatment schedule reported in Figure 3B [31,32,33]. The engraftment with *Sting* wild-type and knockout LLC1-HER2 cells was likewise efficient, without significant differences in tumor growth in untreated animals (Figure 3C,D). 

As for the oncolytic virus, anti PD-1 antibody was also completely ineffective in this tumor model [32,33,34]. The combination of oncolytic R-LM113 and anti PD-1 antibody resulted in 50% of complete tumor eradication, since 4 out of 8 mice were tumor-free by the end of the treatment, in full agreement with the previously reported literature (Figure 3C) [35]. In the same aforementioned established tumor setting, mice bearing subcutaneous LLC1-HER2_SKO tumors were treated with PD-1 blocking antibody as a single agent or in combination with intratumoral injection of R-LM113. As for *Sting* wild-type, anti PD-1 antibody treatment was ineffective, per se, in *Sting* knockout tumors (Figure 3D). The absence of *Sting* expression in tumor cells completely abrogated the effectiveness of combination therapy; in fact, none of the treated mice were tumor free by the end of the treatment (Figure 3D). Interestingly, in half of the animals, the OV treatment significantly delayed the tumor growth compared to matched untreated animals (Figure 3E). This partial response is probably the result of tumor debulking elicited by the lytic function of R-LM113. 

Since oncolytic HSV-1 induces the immunogenicity of tumors, we hypothesized that the loss of *Sting*-dependent antiviral responses might have impaired the proinflammatory phenotype and the tumor immune remodeling. To address this hypothesis, a gene expression profiling was carried out by NanoString PanCancer Immune Profiling and PanCancer Mouse Pathway on samples from mock-treated and virus-injected tumors of both wild-type and *Sting* knockout derivations. In the case of the *Sting* wild-type tumors, most of the differentially regulated genes were upregulated in response to the infection. These genes were predictive of an antitumor immune response signature and matched previous reports in the literature, highlighting the significance of these upregulated pathways [28,31,36]. According to STRING gene ontology (GO) and manual annotation, most of the genes were involved in different facets of T cell response, comprising: activation (*Lat*, *Rorc*), cytotoxic activity (*Prf1*, *Gzma*) and trafficking (*Flna*, *Epha2*); immune checkpoint modulators (*Icos*, *Pd-l2*, *Ctla-4*); and innate immunity activators (*Klrg1*, *Ccl19*, *Txk*, *Id2*) (Figure S7).

**Figure 4:**
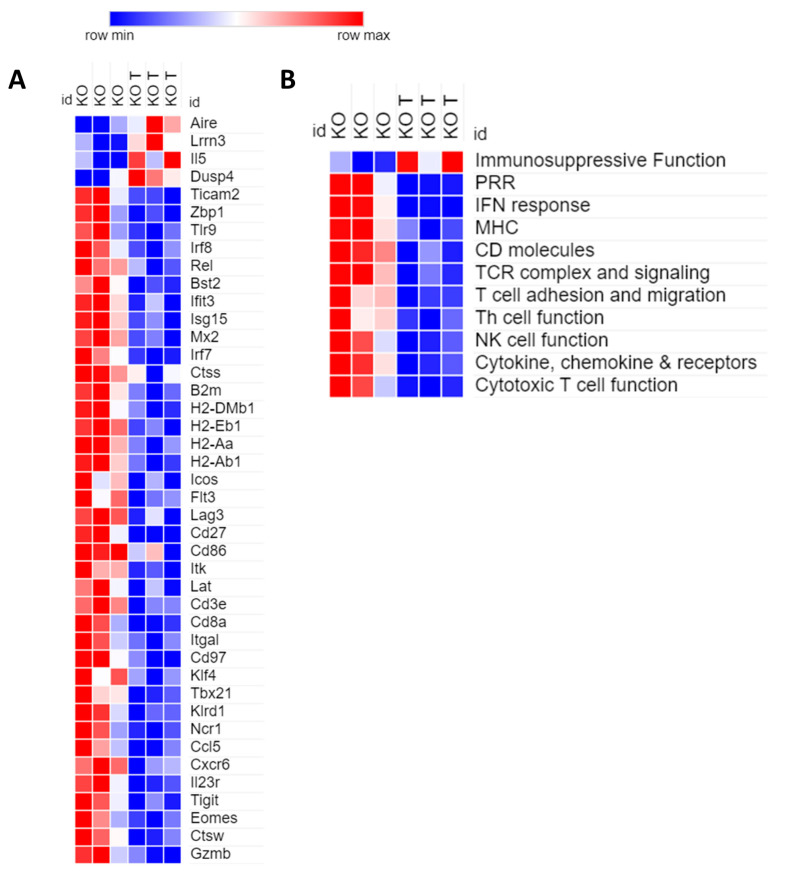
*Sting* loss affects immunogenic tumor remodeling according to NanoString RNA profiling. (**A**) The panel reports the heat map for representative differentially regulated genes between *Sting* knockout untreated (KO) and treated (KO T) tumors. Values were normalized by nSolver software and filtered according to *p*-value (<0.05) and fold (±1.5). (**B**) The genes were grouped in 11 immune-relevant categories to obtain an overview of the gained trend from NanoString analysis.

On the contrary, the transcriptomic profile of *Sting* KO tumors was characterized by general downregulation of immune-related genes as a consequence of oncolytic treatments (Figure 4). Among the few upregulated genes there were: markers used to define resting cytotoxic T cells and predictor of short-term survivors (*Lrrn3*) [37]; oncogenes (*Etv4*) [38]; and *Dusp4*, recently described as a negative regulator of *STING* and *RIG-I* pathway cascade [39]. On the contrary, downregulated genes were classified according to gene ontology in: PRRs (*Rig-I*, *Zbp1*, TLRs, *Oas2*, *Oas3*, *Ifih1*); IFN response; antigen presentation (MHCs, *B2m*); T cell function (TCR signaling, cytotoxicity, adhesion and migration, T helper cell function); NK cell function; cytokines, chemokines and receptors (Figure 4). Figure 4A reports the heat map of representative genes, whereas the full list of differentially regulated ones is reported in the Figure S8. These genes were strongly clustered by STRING analysis, that also revealed the presence of cellular networks involved in the responses to viruses and, specifically, to HSV (Figure S8). The downregulation of herpesvirus-restriction factors (*Bst2*, *Stat2*, *Mx2*) that are also reported to be directly inactivated by HSV-1 is also noteworthy [40,41,42].

### 2.4. STING-Deficient Tumor Cells Do Not Trigger Type I IFN Cascade and Show Impaired Immunogenic Cell Death Responses

To address how *Sting* inactivation in tumor cells might be responsible for impaired immunogenicity of tumor microenvironment, we took into consideration the two key events of antitumor vaccine activity typically exerted by oncolytic viruses: type I IFNs triggering and immunogenic cell death (ICD). 

**Figure 5:**
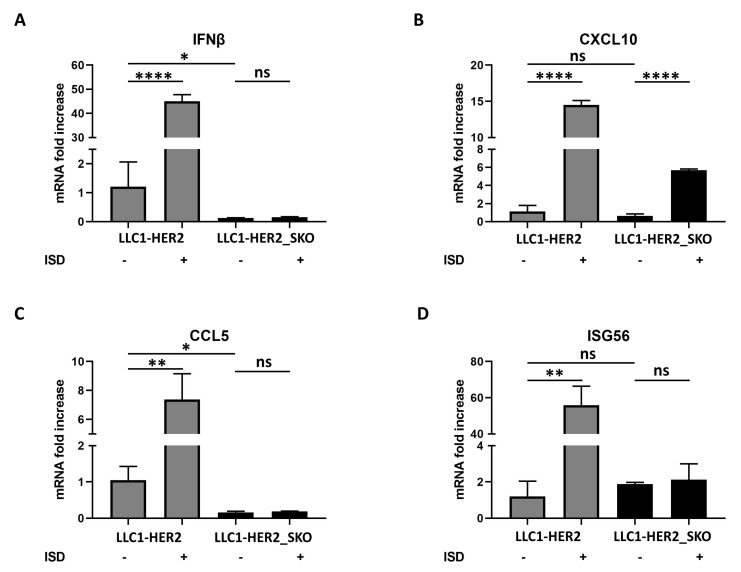
Induction of IFN-I cascade by DNA sensing in Sting knockout and parental cancer cell lines. LLC1-HER2 cells and *Sting* knockout counterparts were stimulated in vitro by interferon stimulatory DNA (ISD). Ten hours post treatment, Ifnb (**A**), Cxcl10 (**B**), Ccl5 (**C**) and Isg56 (**D**) transcripts were assessed by real-time PCR. The relative abundance of target RNAs was evaluated in relation to Actinb transcript. The statistical significances for experiments described in Figure 5 were calculated by Student’s *t*-test. Panel A, the *p*-values were 1.2E-5 comparing untreated and treated LLC1-HER2 and 0.01 comparing *Sting* wild-type vs. knockout cell lines. Panel B, the *p*-values were 1.2E-5 comparing untreated and treated LLC1-HER2 and 3E-6 comparing untreated and treated LLC1-HER2_SKO. Panel C, 0.003 comparing untreated and treated LLC1-HER2; 0.015 comparing Sting wild-type vs. knockout cell lines. Panel D, 0.0008 comparing untreated and treated LLC1-HER2. Ns indicates statistically not significant differences calculated by Student’s *t*-test. *p* <0.05 *; *p* <0.005 **; *p* <0.00005 ****.

LLC-HER2 cells and their *Sting*-knockout counterparts were stimulated in vitro by interferon stimulatory DNA (ISD) and IFN cascade triggering was assessed 10 h post treatment. In *Sting* wild-type cells, the transcription of both direct *Sting* targets and IFN-stimulated genes (*Ifnb*, *Ccl5*, *Isg56*, *Cxcl10*) was strongly upregulated after stimulus (Figure 5). In *Sting*-knockout cells, the basal transcription of IFN-related genes was already dampened, compared to *Sting* wild-type cells, probably as the result of the predicted loss of sensing genomic instability consequent to *Sting* ablation [18]. After stimulation, the transcription upregulation did not occur for three out of the four targets, as only *Cxcl10* was slightly upregulated, though to a lower extent compared to wild-type cells (Figure 5). Similar results were obtained in the CT26 cell line pointing out the central role of antiviral immunity through PRR activation (i.e., *Sting*), regardless of the genetic background (Figure S9).

**Figure 6:**
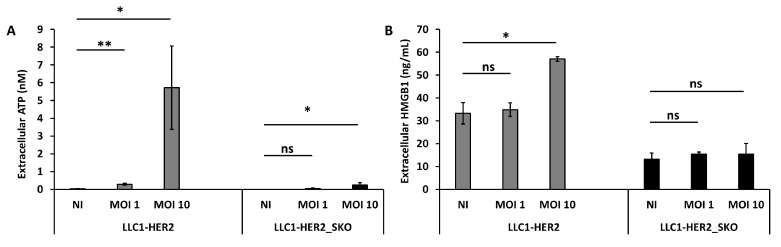
*Sting* expression in tumor cells is essential to induce oncolytic virus-mediated immunogenic cell death. Evaluation of extracellular ATP (**A**) and HMGB1 (**B**) released in supernatant of mock or OV (oncolytic virus)-infected LLC1-HER2 and *Sting* knockout cells. Viral doses are indicated in each panel (1 and 10 PFU/cell). Infections were performed as biological replicates. The statistical significances for experiments described in Figure 6 were calculated by Student’s *t*-test. Panel A, the *p*-values were: 0.0008 comparing untreated and 1 MOI LLC1-HER2; 0.01 comparing untreated and 10 MOI LLC1-HER2_SKO. Panel B, the *p*-value was 0.0199 comparing untreated and 10 MOI LLC1-HER2. Ns indicates statistically not significant differences calculated by Student’s *t*-test. *p* < 0.05 *; *p* < 0.005 **.

In order to understand whether *Sting* also contributed to the elicitation of immunogenic cell death, the release of extracellular ATP and high-mobility group box 1 (HMGB1) were evaluated. LLC-HER2 and the derived *Sting*-knockout cells were infected with R-LM113 at different MOIs (1 and 10) and the selected DAMPs were dosed from conditioned media. As reported in Figure 6, the infection mediated a dose-dependent ATP and HMGB1 release from *Sting* wild-type cells. As for the IFN-related gene expression shown in Figure 5, the basal DAMPs release in mock-infected cells was inhibited in *Sting*-knockout cells compared to the wild-type counterparts. Moreover, despite the improved cell lysis assessed by LDH release (Figure 2), the infection failed to induce ICD in *Sting*-knockout cells. Similarly, in the CT26 cellular background, the absence of *Sting* harmed OV-induced ICD (Figure S10). These data shed light on *Sting* involvement in regulating immunogenicity of cell death, as its loss induces a more tolerogenic cell death, characterized by low release of immunogenic molecules (i.e., ATP, HMGB1), despite the consistent passive LDH release [43].

## 3. Discussion

Oncolytic viruses are a new class of immunotherapeutics with emerging potential as synergistic agents in combination therapies with checkpoint modulators [3,44]. Their multimodal way of action is far from being fully elucidated, which is why a debate is still open between the oncolytic-centric and immune-centric points of view, that consider, respectively, tumor cell lysis and immunogenicity as the key activity of oncolytic viruses [45].

Since the oncolytic-centric faction considers the viral replication and cancer cell clearance as essential, the host antiviral machinery is accounted as a mechanism to be overcome. In this field, with particular regard to DNA-based viruses (i.e., HSV-1), antiviral activity of the *cGAS/STING* axis represents the main hurdle for viral spread and cancer cell killing. Accordingly, therapeutic strategies are currently being explored to optimize more virulent oncolytic viruses and counteract host antiviral response [34].

On the other hand, increasing evidence demonstrates the ability of OVs to induce a T-cell-based antitumor response that is fully represented by occurrence of the abscopal effect [45]. This effect relies on triggering the host antiviral immune response, induction of immunogenic cell death and release of tumor antigens. Emblematic for the immune-centric point of view are those investigations that negatively correlate viral replication to cancer cell immunogenicity and in vivo efficacy [6,45,46,47].

In this tug of war, *STING* is the keystone to dissect oncolytic virus functions and the role of host antiviral immunity. Indeed, experimental evidence has demonstrated both the direct relationship between *STING* loss in tumor cells and OV-mediated cell lysis, and its tumor-extrinsic ability to activate immune cells [20,21,22,24,47]. The tumor-extrinsic function of *STING* is particularly relevant in APCs that are able to trigger a *STING*-dependent type I IFNs cascade by capturing tumor-derived cGAMP or phagocyting tumor cells containing cytosolic DNA stimuli, including oncolytic HSV-1 [19,22].

Considering the aforementioned evidence, the loss-of-function in *STING*-mediated DNA sensing that occurs in tumor cells to escape immune surveillance is thought to represent the Achilles’ heel that makes cancer an exquisite target of oncolytic viruses [24,25,26,27,48]. These speculations have been addressed in xenografts in nude mice which, however, do not keep under consideration the tumor-resident function of *STING* in inducing an antitumor immune response that is still a limitedly explored topic [25,26].

To figure out the actual role of tumor-resident STING within the oncolytic framework of cancer therapy, we generated *Sting* knockout tumor cell lines from C57BL/6 and BALB/c murine genetic backgrounds. In vitro replication and cytotoxicity recapitulated well the *Sting* loss-dependent improved susceptibility of cancer cells to OV already reported in the literature [25,26,27]. On the contrary, the efficacy of R-LM113 and PD-1 blockade combination therapy resulted in 50% and no cured mice, respectively, for *Sting* wild-type and knockout tumors, although the hosting mice were in both cases of a wild-type *Sting* background. Interestingly, *Sting* wild-type tumor-bearing mice showed a clear distinction between complete responders and non-responders, spurring us to further investigate the mechanism of resistance and to develop novel multi-cytokine armed oncolytic HSV-1 to overcome this hurdle [32]. Transcriptomic profiling of *Sting* wild-type vs. knockout tumors showed that in case of *Sting* loss, immunosuppressive function of HSV-1 dominates the host antiviral machinery, which implies keeping an immunosuppressive TME in the presence of OVs. Consonant to in vivo efficacy, RNA profiling data were: (i) prognostic of adaptive antitumor immune response for *Sting* wild-type tumors; (ii) predictive of immunosuppressive TME for *Sting* knockout tumors. In fact, in *Sting*-deficient tumors the antiviral pathways, the components of the antigen-presenting machinery and T cell functions were downregulated. 

In this paper we showed, for the first time, that *Sting* is essential for OV-mediated immunogenic cell death of cancer cells. Indeed, despite improved cell lysis, *Sting* knockout cells succumbed to R-LM113 in an immunologically silent way. That was true not only for poorly immunogenic cell lines (i.e., LLC1) but also for CT26 cells that are considered more immunogenic [30]. 

Our system of *Sting* knockout tumors hosted in mice with a *Sting/cGas* wild-type background allowed us to not neglect the tumor-extrinsic function of *Sting* in APCs, that are likewise able to “sense” viral DNA. Indeed, even if APCs cannot be infected by the retargeted R-LM113 due to entry restriction, they are still able to activate their own STING by: (i) phagocyting viral particles; (ii) engulfment with fragments of dead tumor cells containing viral genomes [22]; and (iii) drawing cGAMP from infected cancer cells via gap junctions [19]. Taking together these considerations, tumor-resident *Sting* emerged as a key bridge between innate antiviral and adaptive antitumor immunity.

Finally, based on the STING-dependent susceptibility of mammalian cells to HSV-1 and additional viruses, it can be predicted that genetic variants of *STING* could contribute to human susceptibility to viral infections by both DNA and RNA viruses [13,25,26,27]. In particular, future efforts could reveal the susceptibility of human cells carrying *STING* variants to the coronavirus SARS-CoV-2 (formerly 2019-nCoV) which is currently responsible for the COVID-19 pandemic.

## 4. Materials and Methods

### 4.1. Cell Culture, Manipulation and Characterization

LLC1-HER2 and LLC1-HER2_SKO were cultured in Dulbecco’s Modified Eagle Medium (DMEM) supplemented with 2 mM L-glutamine; SKOV3, CT26, CT26-HER2, CT26-HER2_SKO were cultured in RPMI 1640 Medium GlutaMAX™ Supplement. All media were supplemented with 10% FBS and Pen/Strep. Puromycin was used for human ERBB2 transgene stable expression. All the reagents were from Gibco^TM^, Thermo Fisher Scientific Waltham, MA, USA. Cell lines were purchased from the American Type Culture Collection (ATCC) or kindly donated from collaborators and cultured in a humidified atmosphere containing 5% CO_2_ at 37 °C. The impact of *Sting* knockout on viral replication was at first evaluated in a pilot study carried out on cells generated by standard homologous recombination targeting exons 3 and 4 of *Sting*. To avoid in vivo immunogenicity of antibiotic resistant genes, *Sting* knockout was again carried out by CRISPR/Cas9 using pSpCas9(BB)-2A-GFP (PX458 ADDGENE, Watertown, MA, USA) with gRNA reported in Table 1. Knockout and functional rescue were assessed by Western blot [49]. Filters were probed with the anti-STING antibody (Cell Signaling, Danvers, MA, USA, #13647), followed by anti-rabbit secondary antibody. Pierce™ ECL Western Blotting Substrate (Thermo Scientific, Waltham, MA, USA) was used for signal development, according to the manufacturer’s recommendations. Human *HER2* transduction of CT26 cells was performed by Origene, Rockville, MD, USA, RC212583L1V. To evaluate the HER2 expression, cells were stained with FITC-conjugated anti-human HER2 (ab31891 Abcam, Cambridge, UK) and analyzed with FACS.

### 4.2. Cytotoxicity Assay

The lysis of virus-infected cells was determined by measuring the release of extracellular lactate dehydrogenase (LDH) from cells infected with R-LM113 at different MOI (1 pfu/cell and 5 pfu/10 cell) over mock-infected cells by Pierce LDH Cytotoxicity Assay Kit (Thermo fisher Scientific, Waltham, MA, USA).

### 4.3. Virus Production, Titration and Real Time PCR Analysis

The R-LM113 virus used in this article was described in Menotti el al. [29]. The virus was produced and titrated in SKOV3 cells according to the procedure previously described [29]. To analyze the viral replication, genome copies were titrated by TaqMan RealTime PCR (Taqman universal PCR mastermix, Applied Biosystems, Foster City, CA, USA) from cell lysates. Briefly, viral samples were diluted in A195 buffer and treated with RNase-free, DNase I recombinant enzyme (Roche, Basel, Switzerland) to remove envelope-free viral DNA. Enveloped viral DNA was thus extracted by SDS 0.1% (w/v, final concentration) and proteinase K (Roche, Basel, Switzerland). The extracted viral particles were diluted 1:10, 1:100 and 1:1000 and analyzed by TaqMan RealTime PCR according to the manufacturer’s recommendations (oligoes and probe in Table 1).

### 4.4. In Vivo Studies and Ex Vivo Genome Copies Analysis

Female heterozygous B6.Cg-Pds5b<Tg(Wap-ERBB2)229Wzw>/J mice were used for in vivo studies [35]. Mice were implanted subcutaneously on the right flank with 5 × 10^5^ LLC1-HER2 or LLC1-HER2_SKO cells. Ten days after challenge, mice bearing established tumors were randomized according to tumor size, and 1E+08 viral PFU were injected intratumor in combination with intra-peritoneally treatment with 200 μg α-mPD-1 (BioXcell, clone RMP114). The growth of tumors was measured by caliper every 3 or 4 days using the formula (LxW2)/2 [50]. Animals were sacrificed as soon as signs of distress or a tumor volume above 1500 mm^3^ occurred. In vivo viral replication was assessed 48 and 72 h after a single dose injection of 1E+08 viral PFU by TaqMan PCR. The experimental procedures were approved by the Italian Ministry of Health (Authorizations 213/2016 PR)

### 4.5. NanoString Data

Mice were implanted subcutaneously on the right flank with 5 × 10^5^ LLC1-HER2 or LLC1-HER2_SKO cells. Ten days after challenge, mice bearing established tumors were randomized according to tumor size and treated with 1E+08 viral PFU intratumor injection or untreated. After 24 h, the tumors were harvested, collected in RNA later (QIAGEN, Hilden, Germany) and stored at − 80 °C. Tumors were lysed by Tissue Lyser LT (QIAGEN, Hilden, Germany) with 5 mm beads (QIAGEN, Hilden, Germany) in the presence of 2-mercaptoethanol (INVITROGEN, Carlsbad, CA, USA). To extract total RNA, an Rneasy Mini kit (QIAGEN, Hilden, Germany) was used. The extracted RNAs were analyzed by nCounter Mouse PanCancer Immune Profiling Panel, in which were examined 770 immune-related genes, and Mouse PanCancer Pathways Panel, where we examined 770 genes belonging to 13 cancer-associated canonical pathways. Data were processed and normalized using nSolver Analysis Software.

### 4.6. In Vitro mRNA Dosage 

IFN response-related genes were evaluated by using quantitative RT-PCR. Briefly, at day -1 LLC1-HER2, CT26-HER2, LLC-HER2_SKO and CT26-HER2_SKO cells were seeded in 12-well plates; at day 0 cells were transfected with 3 μg of interferon stimulatory DNA (ISD) (Invivogen, San Diego, CA, USA) in a ratio of 1:1 DNA/lipofectamine or with only lipofectamine 2000 (Invitrogen, Carlsbad, CA, USA). Ten hours after transfection, cells were lysed by TriFast (Euroclone, Pero, Italy) and total RNA was extracted with phenol/chloroform. Then, 3 μg of RNA was treated with RQ1 RNase-free DNase (Promega, Madison, WI, USA) to eliminate residual DNA contaminants. After Dnase inactivation for 10 min at 65 °C, 1 μg of RNA was reverse-transcribed by using ImProm-II Reverse Transcriptase (Promega, Madison, WI, USA) in a mix containing 3 mM MgCl_2_, 0.5 mM dNTP and 500 ng random primer (Invitrogen, Carlsbad, CA, USA). The cDNA was then amplified in a 7500 Real-Time PCR System (Applied Biosystem, Foster City, CA, USA) using SYBR Green PCR Mastermix (Applied Biosystem, Foster City, CA, USA) as reported in previous paper [33]. All oligonucleotide primers were used to a final concentration of 0.2 µM (Table 1). The relative abundance of target RNAs was evaluated in relation to β-actin transcript by ΔΔCt method [33]. 

### 4.7. Immunogenic Cell Death

Immunogenic cell death mechanism was investigated through the extracellular release of ATP and High Mobility Group Box 1 (HMGB1). LLC1-HER2, CT26-HER2, LLC-HER2_SKO and CT26-HER2_SKO cells were seeded in 12-well plates and infected with R-LM113 at 1 and 10 pfu/cell or mock-infected. The supernatants were collected 24 h post infection and debris were removed by centrifugation at 200× *g* for 5 min. Secreted ATP was measured by ENLITEN ATP Assay System (Promega) according to the manufacturer’s protocol. Supernatants were also used to detect HMGB1 with HMGB1 ELISA Kit (IBL International, Hamburg, Germany) following the manufacture’s protocol outlined for the normal sensitivity format of the assay.

### 4.8. Statistical Analysis

GraphPad Prism was used to perform the following statistical analysis: Student’s *t*-test, two-way ANOVA and Fisher’s exact test. The significance was reported according to the following code *p* < 0.05 *; *p* < 0.005 **; *p* < 0.0005 ***; *p* < 0.00005 ****.

## 5. Conclusions

In short, through activation of *Sting*-dependent antiviral cascade in cancer cells, oncolytic viruses can successfully activate antitumor immunity, satisfying the three requirements for T-cell responses: (i) tumor antigen release and MHC-I presentation [51]; (ii) co-stimulation induced by ICD-activated DCs; (iii) cytokine production [8]. The evidence collected in this paper further underlines that in the explored system the tumor immune remodeling induced by oncolytic virotherapy overcomes the therapeutic effect of oncolytic HSV-1 and supports the antitumor benefit of antiviral immunity [8].

Most importantly, due to the non-redundancies in the pathway, this study emphasizes the importance of conducting a clinical retrospective study to correlate the inactivation of any of the *cGAS*, *STING*, *TBK1* and *IRF3* genes to the clinical outcome of oncolytic virotherapy. In addition, the *STING* gene is highly heterogeneous in populations. Besides the most common R232 human allele, natural variants of *STING* with reduced or null activity have been reported with a considerably high allele frequency. These include H232 and HAQ (R71H-G230A-R293Q) variants that are widespread as homozygous or compound heterozygous in ~30% of East Asians and ~10% of Europeans [21,25]. Based on the evidence reported in this paper, patients with a partial or complete loss-of-function *STING* genotype may not take full advantage of oncovirotherapy. The potential stratification of responder and non-responder patients according to tumor-resident DNA sensing status could represent a milestone to support the identification of patients that are good candidates for oncolytic virotherapy. Moreover, as a low amount of *STING* in tumor cells was described as sufficient to partially trigger OV-mediated immunogenicity [27,28], we aim to implement future strategies to rescue its function in *STING* null tumors as preparatory to OV treatment.

## 6. Patents 

A patent has been filed with some data reported in this manuscript.

## Figures and Tables

**Table 1:** Oligonucleotides.

Name	Oligonucleotide Sequences
Taqman_DNApol_Fwd	5′-catcaccgacccggagagggac-3′
Taqman_DNApol_Rev	5′-gggccaggcgcttgttggtgta-3′
Taqman Probe	FAM-ccgccgaactgagcagacacccgcgc-Tamra
CCL5_RT_Fwd	5′-cctcaccatatggctcggac-3′
CCL5_RT_Rev	5′tcttctctgggttggcacac-3′
CXCL10_RT_ Fwd	5′-gccgtcattttctgcctcatc-3′
CXCL10_RT_ Rev	5′-taggctcgcagggatgatttc-3′
IFIT/ISG56_RT_Fwd	5′-tccgtaggaaacatcgcgtag-3′
IFIT/ISG56_RT_Rev	5′-tcttgcacattgtcctgcct-3′
IFNβ1_RT_Fwd	5′-atttctccagcactgggtgg-3′
IFNβ1_RT_Rev	5′-aggtacctttgcaccctcca-3′
CAS9_Fwd	5′-gctctttgatgccctcttcg-3′
CAS9_Rev	5′-gctgaccctgacactgtttg-3′
GFP_Fwd	5′-cacgacttcttcaagtccgc-3′
GFP_Rev	5′-ggtgttctgctggtagtggt-3′
GuideRNA_1	5′-gaggtcaccgctccaaatat-3′
GuideRNA_2	5′-cacctagcctcgcacgaact-3′
GuideRNA_3	5′-gggatgccccatccactgta-3′

## Supplementary Materials

The following are available online at https://www.mdpi.com/2072-6694/12/11/3407/s1, Figure S1: RNA sequencing analysis of genes involved in cytosolic DNA sensing in CT26 cell line, Figure S2: RNA sequencing analysis of genes involved in cytosolic DNA sensing in LLC1 cell lline, Figure S3: Sting wild-type and KO calls are equally infected by oncolytic R-LM113 at early time point, Figure S4: The knock down of Sting partially restores the replication of oncolytic R-LM113, Figure S5: Comparison of viral effectiveness in Sting knockout vs. parental wild-type cancer cell lines, Figure S6: Functional rescue of STING in CT26-HER2_SKO cell line restored the resistance to oncolytic HSV-1, Figure S7: Gene expression profiling of Sting wild-type LLC1 tumors, Figure S8: Gene expression profiling of Sting knockout LLC1 tumors, Figure S9: Induction of type-I IFN and related genes triggered by DNA sensing in Sting knockout and parental cancer cell lines, Figure S10: STING expression in tumor cells is essential to induce oncolytic virus-mediated Immunogenic Cell Death of cancer cells.
