# Supplementary material for: Integrity of the Antiviral STING-mediated DNA Sensing in Tumor Cells Is Required to Sustain the Immunotherapeutic Efficacy of Herpes Simplex Oncolytic Virus
Source: Cancers (Basel). 2020 Nov 17;12(11):3407. doi: 10.3390/cancers12113407 (PMC7698602; doi:10.3390/cancers12113407)

# Supplementary Materials:

# Integrity of the Antiviral STING-mediated DNA Sensing in Tumour Cells Is Required to Sustain the Immunotherapeutic Efficacy of *Herpes Simplex* Oncolytic Virus

**Guendalina Froechlich, Carmen Caiazza, Chiara Gentile, Anna Morena D'Alise, Maria De Lucia, Francesca Langone, Guido Leoni, Gabriella Cotugno, Vittorio Scisciola, Alfredo Nicosia, Elisa Scarselli, Massimo Mallardo, Emanuele Sasso and Nicola Zambrano**

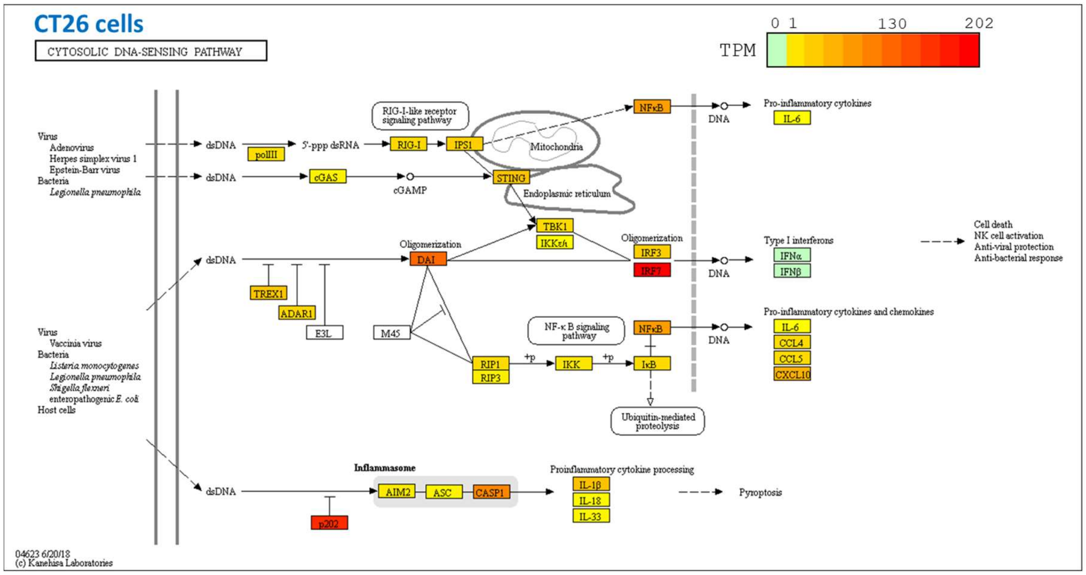


**Figure S1.** RNA sequencing analysis of genes involved in cytosolic DNA sensing in CT26 cell line. Mapping of genes involved in DNA sensing pathways was performed by using the Kegg mapper utility available in Kegg database. The abudance of transcripts were calculated by Transcripts Per Kilibase Million; the gradient of colours, shown in top right box from 202 to 0 TPM, correspond to the different grade of gene expression.

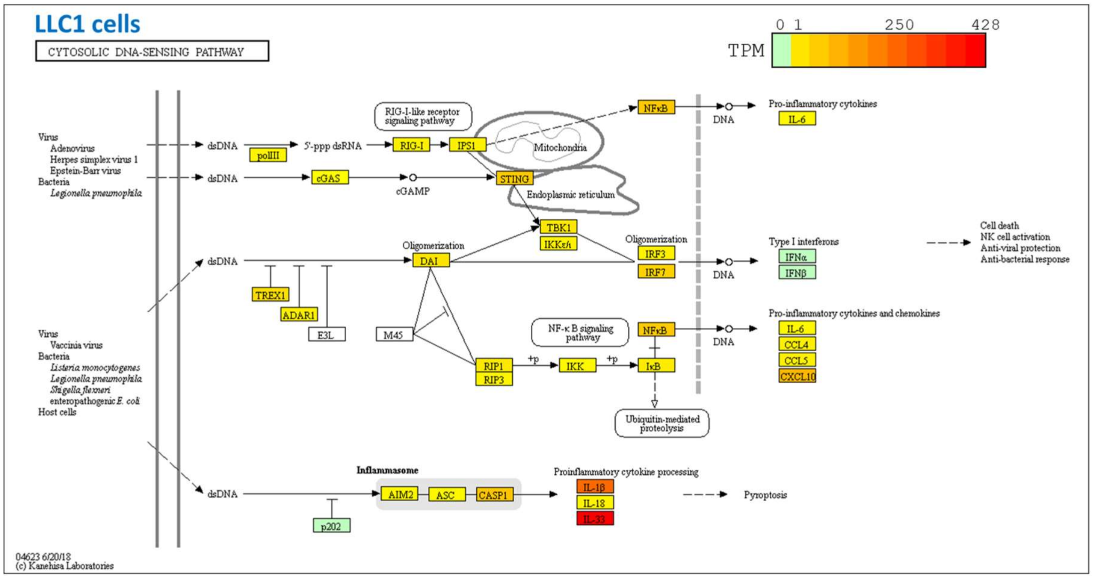


**Figure S2.** RNA sequencing analysis of genes involved in cytosolic DNA sensing in LLC1 cell lline. Mapping of genes involved in DNA sensing pathways was performed by using the Kegg mapper utility available in Kegg database. The abudance of transcripts were calculated by Transcripts Per Kilibase Million; the gradient of colours, shown in top right box from 428 to 0 TPM, correspond to the different grade of gene expression.

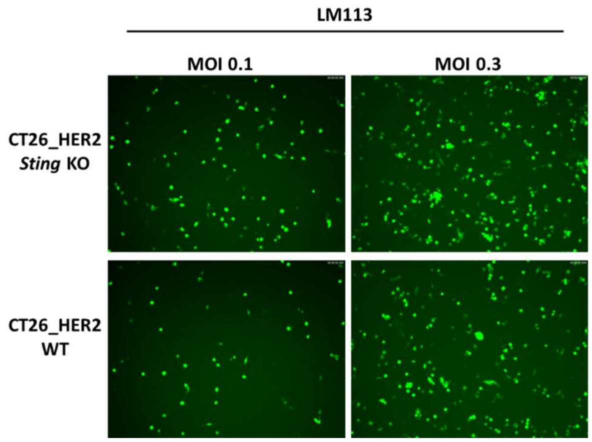


**Figure S3.** Sting wild type and KO calls are equally infected by oncolytic R-LM113 at early time point. Evaluation of viral entry in CT26_HER2 wild type and Sting KO cells by BAC encoded eGFP. Cells were infected at MOI 0.1 and 0.3 and entry efficiency was evaluated 24 h post infection.

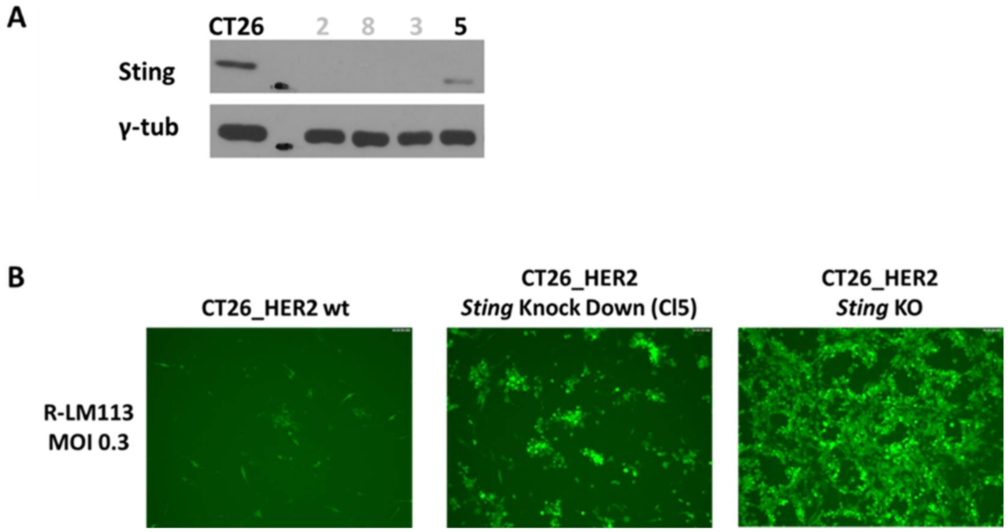


**Figure S4.** The knock down of Sting partially restores the replication of oncolytic R-LM113. (**A**) The expression of Sting protein was evaluated by western blot analysis in different sub-clones. Clone n5 was selected as representative for knock down. (**B**) Evaluation of viral spread in CT26_HER2 wild type, Sting knock down and Sting KO cells by BAC encoded eGFP. Cells were infected at MOI 0.3 and spread efficiency was evaluated 72 h post infection.

Western Blot Original Figure S4A

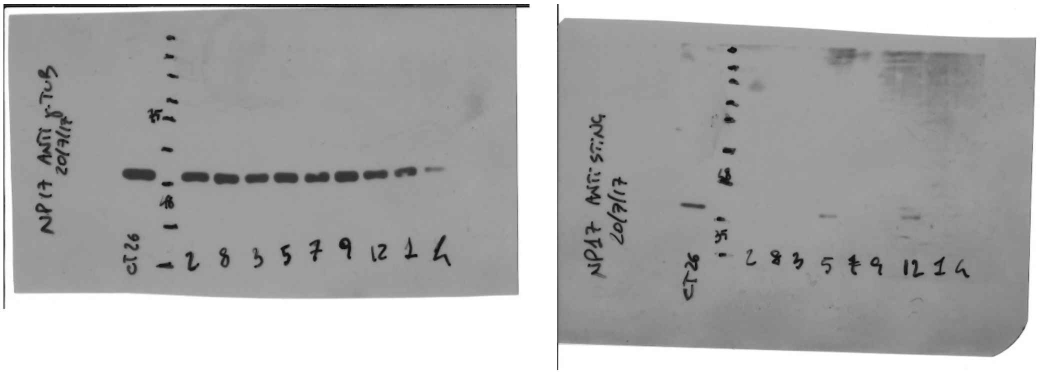

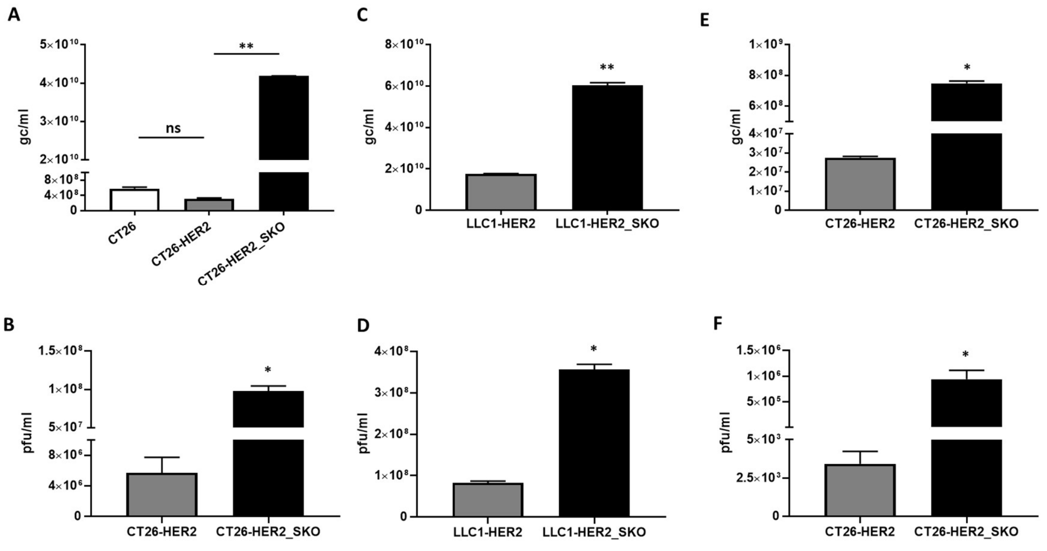

**Figure S5.** Comparison of viral effectiveness in Sting knockout vs parental wild type cancer cell lines. (**A**) Evaluation of viral replication of R-LM55 in CT26, CT26_HER2 and CT26_HER2_Sting knockout cell lines infected with 0.3 PFU/cell. The qPCR-TaqMan analysis revealed the genome copies per mL (gc/mL) produced by the virus at 96 hours post infection. The statistical significance was calculated by Student's t-test comparing Sting wild type vs knockout cell lines, the P value resulted 0.0007. (**B**) Evaluation of the viral titres obtained in Sting wild type and knockout CT26 cells with R-LM55 viruses (0.3 PFU/cell) by plaque assays at 96 hours post infection. The statistical significance was calculated by Student's t-test that resulted 0.03. (**C**,**D**) The same experiments performed in panels **A** and **B** were replicated in LLC1-HER2 and LLC1-HER2_SKO (**C**) and (**D**). The statistical significance was calculated by Student's t-test that resulted respectively 0.0009 and 0.03 for panels **C** and **D**. (**E**,**F**) The CT26 cell lines were infected with R-LM113 with 0.05 PFU/cell. Evaluation of viral genome copies (**E**) and titer (**F**) were performed at 96 hours post infection. The statistical significance was calculated by Student's t-test (0.006 and 0.05 for panel **E** and **F**).

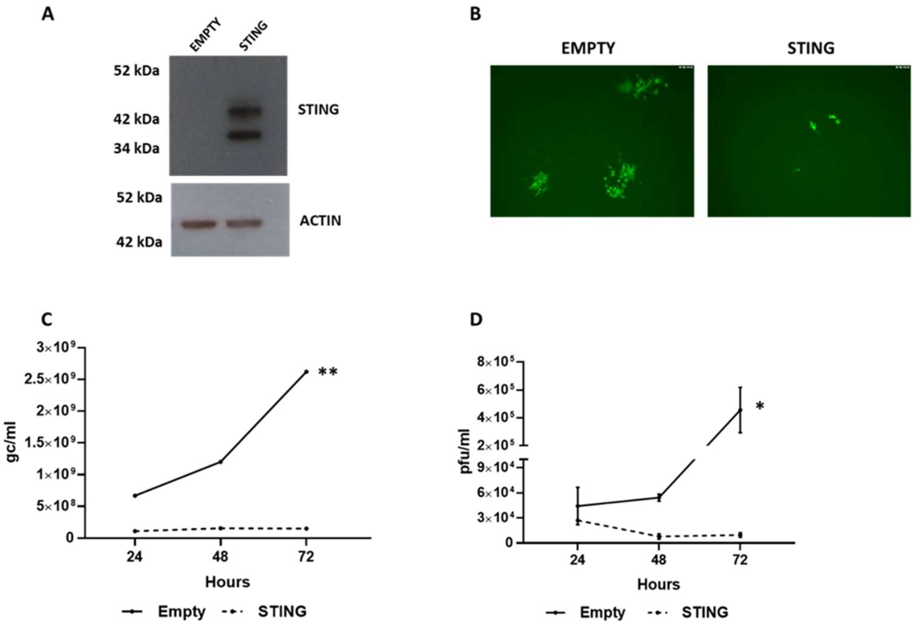


**Figure S6.** Functional rescue of STING in CT26-HER2_SKO cell line restored the resistance to oncolytic HSV-1. CT26-HER2_SKO cell line were transiently transfected with a STING-encoding plasmid and then infected with R-LM113 (0.1 PFU/cell). (**A**) Western Blot analysis of STING protein in mock and Sting-transfected CT26-HER2_SKO; ACTIN was used as standard. (**B**–**D**) Evaluation of: (B) spread of eGFP-encoding R-LM113; viral replication (**C**) and titer (**D**) of R-LM113 in mock and Sting-transfected CT26-HER2_SKO cell line. In panel **C** and **D**, gc and pfu per ml were calculated over time at indicated time points. The statistical significance was calculated by Student's t-test that resulted 0.0023 for viral replication, 0.05 for viral titer.

Western Blot Original Figure S6A

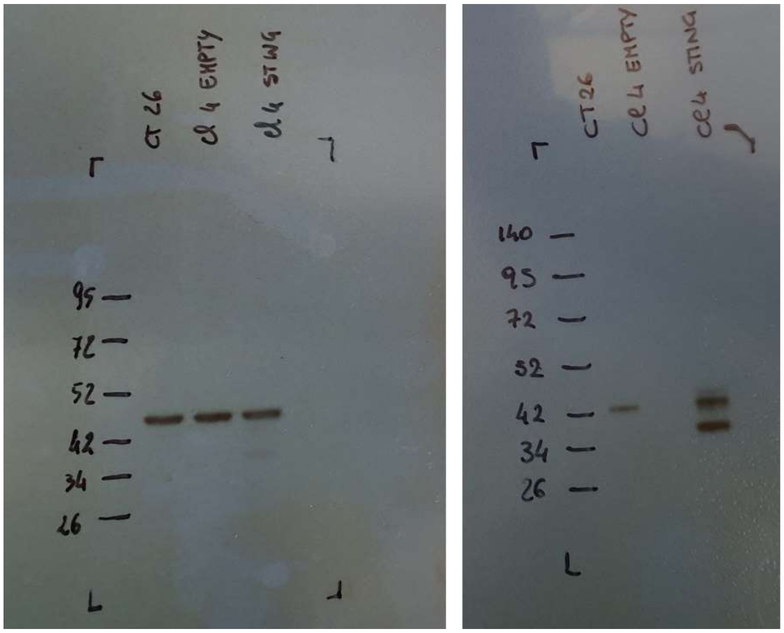


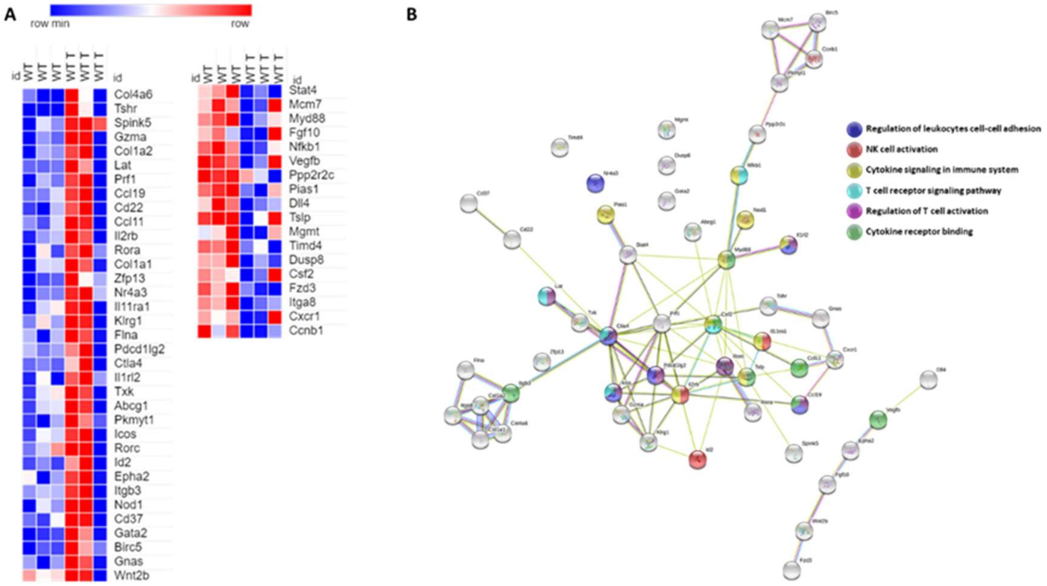


**Figure S7.** Gene expression profiling of Sting wild-type LLC1 tumours. A gene expression profiling was carried out for mock-treated and virus-injected Sting wild type tumours. The image shown the full list of 54 differentially regulated genes as a heat map in panel (**A**) and interaction networks processed by STRING software (**B**). In panel B, genes were labelled according to GO function reported in the picture.

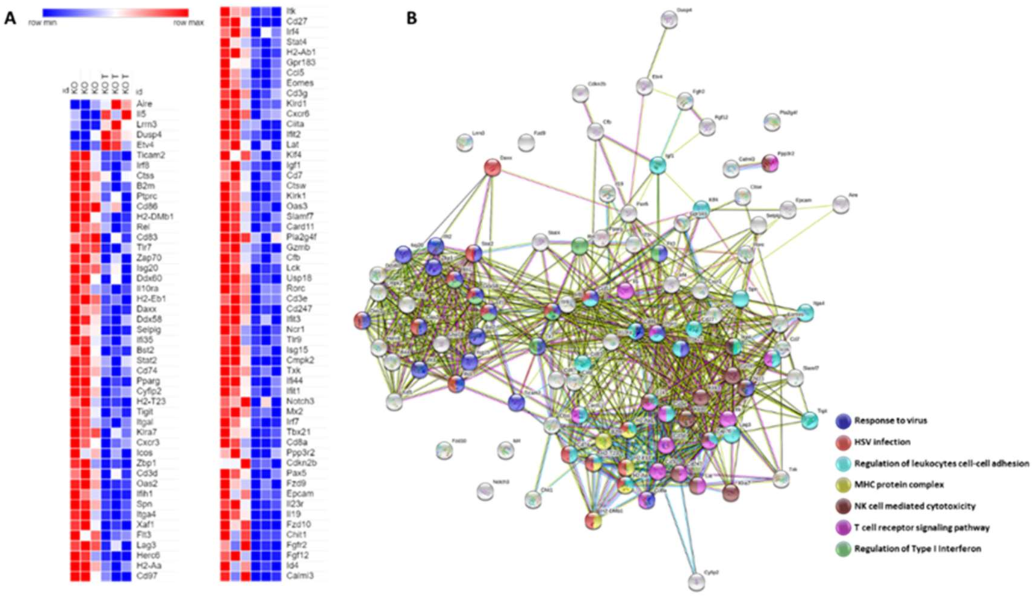


**Figure S8.** Gene expression profiling of Sting knockout LLC1 tumours. A gene expression profiling was carried out for mock-treated and virus-injected Sting knockout tumours. The image shown the full list of differentially regulated genes as a heat map in panel (**A**) and interaction networks processed by STRING software (**B**). In panel B, genes were labelled according to GO function reported in the picture.

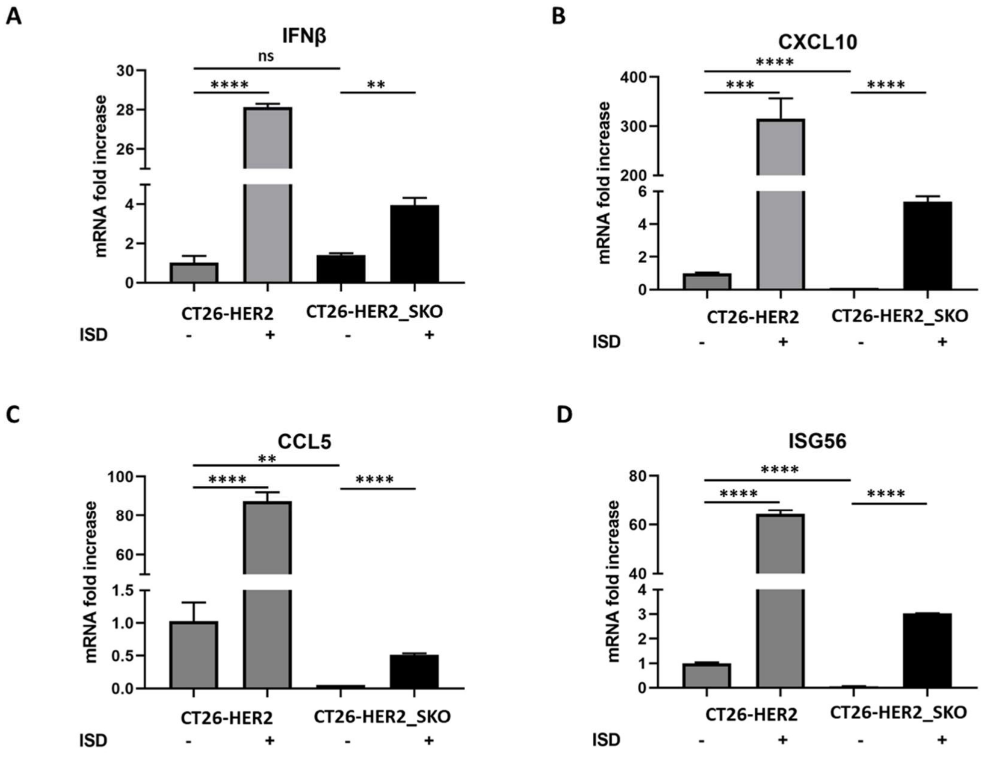


**Figure S9.** Induction of type-I IFN and related genes triggered by DNA sensing in Sting knockout and parental cancer cell lines. CT26-HER2 cells and Sting knockout counterpart were stimulated in vitro by ISD. Transcriptional activation of Ifnb (**A**), Cxcl10 (**B**), Ccl5 (**C**) and Isg56 (**D**) was assessed ten hours post treatment by Real time PCR. Actinb transcript was used to calculate the relative abundance of target genes.

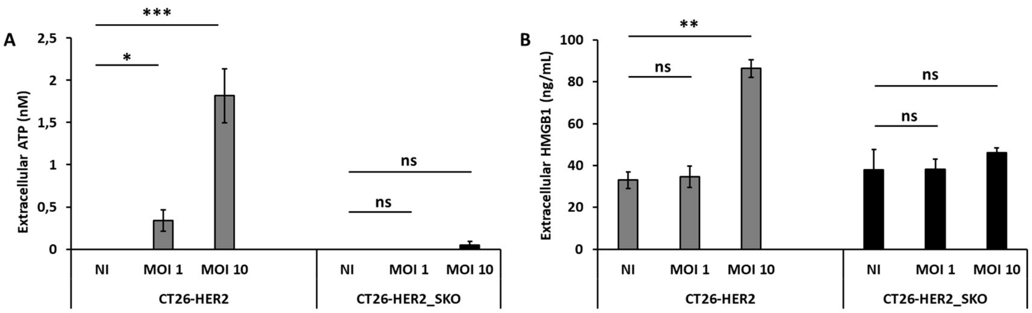


**Figure S10.** STING expression in tumour cells is essential to induce oncolytic virus-mediated Immunogenic Cell Death of cancer cells. Evaluation of DAMP release (extracellular ATP (**A**) and HMGB1 (**B**)) in supernatant of mock or OV-infected (at indicated pfu/cell) CT26-HER2 and Sting knockout cells.

Western Blot Original Figure 1C

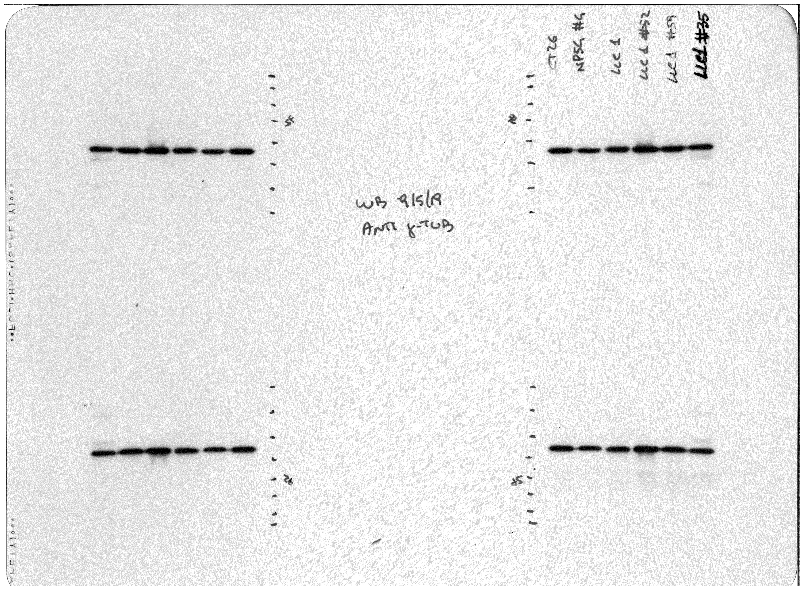

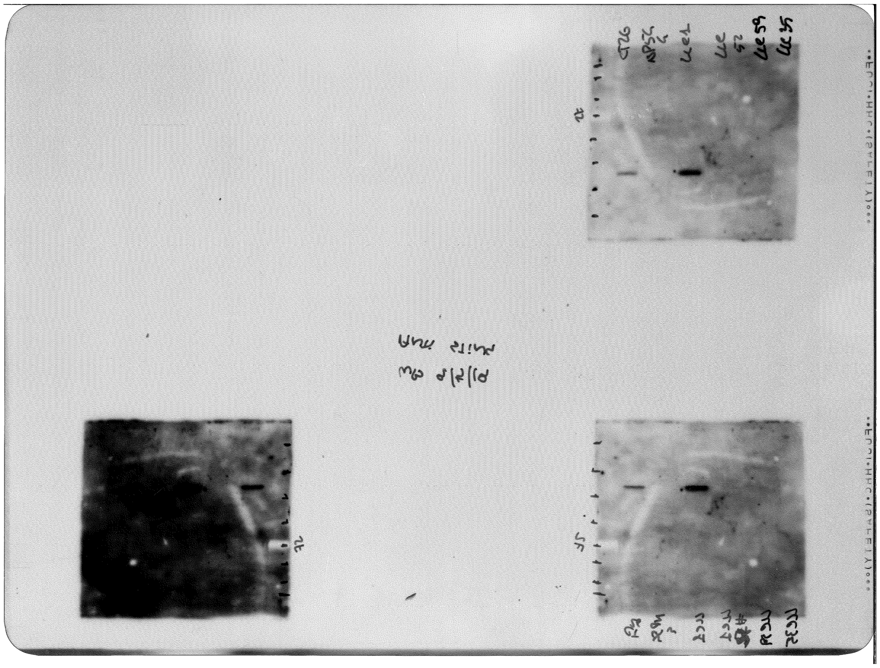

Supplement: Supplementary file 1 [file cancers-12-03407-s001.pdf]
